# *Aedes albopictus* saliva contains a richer microbial community than the midgut

**DOI:** 10.1186/s13071-024-06334-1

**Published:** 2024-06-25

**Authors:** Maria G. Onyango, Anne F. Payne, Jessica Stout, Constentin Dieme, Lili Kuo, Laura D. Kramer, Alexander T. Ciota

**Affiliations:** 1 Department of Biological Sciences, Texas Tech University, 2901 Main St, Lubbock, Texas 79409-3131 USA; 2 New York State Department of Health, Wadsworth Center, 5668 State Farm Road, Slingerlands, NY 12159 USA; 3 School of Public Health, State University of New York Albany, 1400 Washington Avenue, Albany, NY 12222 USA

**Keywords:** *Aedes albopictus*, Saliva microbes, Zika virus, Midgut microbes

## Abstract

**Background:**

Past findings demonstrate that arthropods can egest midgut microbiota into the host skin leading to dual colonization of the vertebrate host with pathogens and saliva microbiome. A knowledge gap exists on how the saliva microbiome interacts with the pathogen in the saliva. To fill this gap, we need to first define the microbial composition of mosquito saliva.

**Methods:**

The current study aimed at analyzing and comparing the microbial profile of *Aedes albopictus* saliva and midgut as well as assessing the impact of Zika virus (ZIKV) infection on the midgut and saliva microbial composition. Colony-reared *Ae. albopictus* strains were either exposed to ZIKV infectious or noninfectious bloodmeal. At 14 ays postinfection, the 16S V3–V4 hypervariable *rRNA* region was amplified from midgut and saliva samples and sequenced on an Illumina MiSeq platform*.* The relative abundance and diversity of midgut and saliva microbial taxa were assessed.

**Results:**

We observed a richer microbial community in the saliva compared with the midgut, yet some of the microbial taxa were common in the midgut and saliva. ZIKV infection did not impact the microbial diversity of midgut or saliva. Further, we identified *Elizabethkingia* spp. in the *Ae. albopictus* saliva.

**Conclusions:**

This study provides insights into the microbial community of the *Ae. albopictus* saliva as well as the influence of ZIKV infection on the microbial composition of its midgut and saliva. The identification of *Elizabethkingia* spp., an emerging pathogen of global health significance, in *Ae. albopictus* saliva is of medical importance. Future studies to assess the interactions between *Ae. albopictus* saliva microbiome and ZIKV could lead to novel strategies for developing transmission barrier tools.

**Graphical Abstract:**

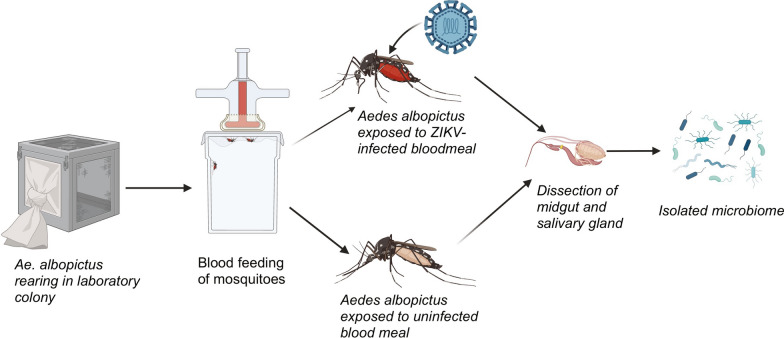

**Supplementary Information:**

The online version contains supplementary material available at 10.1186/s13071-024-06334-1.

## Background

The microbial community in residence in the mosquito midgut plays a key role in determining the outcome of the pathogen infection of mosquitoes [1–8]. Midgut microbiota interferes with pathogen infection by activating the basal immunity of the mosquito [3, 4, 9] as well as releasing metabolites that contain antimicrobial properties [10]. Indeed, due to their potential as candidates for the development of transmission-blocking agents, investigators have studied the interaction of gut microbial taxa and arboviruses of medical importance extensively [2–4, 11–15]. At the field application level, *Wolbachia*, a maternally inherited intracellular bacteria, has demonstrated significant success in containing the spread of dengue and Zika on massive scales [16, 17]. Evidence shows the activation of mosquito immune responses by *Wolbachia* subsequently limits viral replication [18].

Midgut microbiota is also present in the arthropod saliva and can be egested at the skin bite site [19–21]. Indeed, past findings have demonstrated that the sandfly midgut microbiome is egested into the host skin together with *Leishmania* parasites [19]. The midgut microbiota egested on the host skin triggers the inflammasome to produce Interleukin-1 beta (IL-1β), a cytokine whose main role is inflammation and immune-amplifying effects, resulting in sustained recruitment of neutrophils. The neutrophils shield the *Leishmania* parasites thus enhancing parasite visceralization and disease severity [19]. The same study demonstrated that the reduction of midgut microbiota by pretreatment of *Leishmania*-infected sandflies with antibiotics results in a reduction in neutrophil recruitment and impairment of parasite visceralization [19]. Taken together, the egestion of arthropod-borne microbiota at the bite site may be a conserved mechanism [19], yet there is a lack of knowledge on how the mosquito saliva microbiome interacts with arthropod-borne viruses contained in the saliva. This knowledge is important as it can lead to an improved understanding of the initial stages of virus transmission from the mosquito saliva to the human host and the identification of potential targets for transmission barrier development. However, before these studies can be conducted, we need to gain a greater understanding of the bacterial taxa composition of the mosquito saliva.

Indeed, mosquito saliva is important in aiding the replication and dissemination of viruses in vertebrate hosts. In a separate study, *Ae. aegypti* mosquito bites followed by injection of a known viral titer of Semliki Forest virus (SFV) and Bunyamwera virus (BUNV) inoculums resulted in a significantly higher virus RNA copy number in the skin at the inoculation site compared with unbitten mice that were injected with similar virus titer [22]. The study concluded that mosquito saliva augmented virus replication in the myeloid cells and dissemination leading to a more severe outcome. The myeloid cells were attracted to the bite site by a neutrophil-driven inflammasome-dependent skin bite site edema inflammation. Neutrophil depletion resulted in repressed inflammation and the inability of the mosquito bite to enhance viral replication [22]. Furthermore, recent reports demonstrate that Anopheline mosquito saliva contains bacteria that are transferred to a mammalian host through blood feeding [20]. The report demonstrated a successful dual colonization of mammalian host tissues by bacteria and *Plasmodium berghei* transmitted through the *Anopheles* spp. mosquito saliva [20]. In addition, *An. gambiae* infected with *Rickettsia felis* causes a systemic infection in mice [21].

*Ae. albopictus* (Skuse) (Subgenus: *Stegomyia*; *Diptera*: *Culicidae*) is a medically important vector of viruses that causes diseases of major global public health concerns and economic burdens such as dengue virus (DENV), chikungunya virus (CHIKV) and Zika virus (ZIKV) [23, 24]. It is quite invasive, globally spread to over 129 countries [25]. *Ae. albopictus* demonstrates high ecological plasticity, is evenly distributed in suburban, rural, and sylvatic habitats, and can utilize both clean and stagnant water to hatch its eggs [26]. Other than the pathogens that they transmit, mosquitoes harbor other microorganisms such as bacteria [26–28]. Consumption of water, nectar, or other environmental food sources acts as a source of microbiota associated with *Ae. albopictus *[29–31]. The microbial composition of *Ae. albopictus* differs across tissues, life stages, individuals, and geographical regions [32, 33]. In addition, past studies demonstrate that viral infection of the mosquito midgut impacts its dynamic bacterial community. ZIKV infection, for instance, reduces the *Ae. albopictus* midgut microbial diversity[34], while infection with La Crosse orthobunyavirus lowers the midgut microbial richness of *Ae. japonicus* and *Ae. triseriatus *[35]. Lastly, exposure of *Culex pipiens* to West Nile Virus infection results in a lower bacterial diversity [36]. Despite past findings on the significant impact of viral infection on the microbiome composition of the mosquito midgut, the effect of viral infection on the mosquito saliva microbiome community is poorly studied. In the present study, we hypothesized that there are conserved bacterial taxa in the saliva and midgut of *Ae. albopictus* and that ZIKV infection may alter the microbial composition of *Ae. albopictus* saliva and midgut. We tested our hypotheses by analyzing the microbial profile of *Ae. albopictus* midgut and saliva exposed to a ZIKV-infected and uninfected blood meal. In the future, our findings can be built upon to understand the interaction of *Ae. albopictus* saliva microbiome and ZIKV. Further, our findings can be utilized as an avenue for an innovative approach to develop a transmission-blocking tool.

## Methods

### Virus

The ZIKV strain utilized in this study was HND (2016-19563, GenBank accession no. KX906952). The Zika virus isolate used in this study was obtained from an infected patient serum. In the lab, virus stocks were created by passaging three times on Vero cell culture (ATCC) and once on C636 cell culture (ATCC). Stocks were frozen at −70 °C before use.

### Insect sampling and sample preparation

*Ae. albopictus* mosquitoes were collected from Suffolk County, New York State (NYS) in 2015 (kindly provided by I. Rochlin) and colonized at the New York State Department of Health (NYSDOH) Arbovirus Laboratory. The F15 generation *Ae. albopictus* eggs were vacuum hatched. The larvae that emerged from the hatched eggs were maintained in plastic rectangular flat containers (35.6 cm length × 27.9 cm width × 8.3 cm height; Sterilite, catalog no. 1963) at a density of one larva per 5 ml of dechlorinated water and reared at 60% relative humidity and a light–dark (LD) photoperiod of 14:10 h. The larvae were fed 1.25 mg/larvae of Tetra Pond Koi growth feed for first and second instar larvae and 2.5 mg/larvae for third and fourth instar larvae [37]. Upon emergence, the male and female adults were transferred to 3.8-l containers and housed together for 8 days at 28 °C at a relative humidity of 60% while being provided with sugar and water ad libitum [37]. To stimulate blood feeding, females were starved of water and sugar for 12 h before infectious blood meal according to our insectary laboratory standard operating procedures. The blood meal consisted of either 1:1 dilution of defibrinated sheep blood plus 2.5% sucrose, sodium bicarbonate (to adjust pH to 8.0) and virus, or a non-infectious blood meal containing a final concentration of 2.5% sucrose solution. Infectious blood meals contained 8.3 log_10_ PFU/ml ZIKV HND [38]. The female mosquitoes were exposed to blood meals in a 37 °C preheated Hemotek membrane feeding system (Discovery Workshops, Accrington, UK) with a porcine sausage casing membrane. After an hour, the mosquitoes were anesthetized with CO_2_ and immobilized on a prechilled tray connected to 100% CO_2_ Engorged females were separated and placed in separate 0.6 l cardboard cartons (30 individuals per carton). Blood-fed females were maintained on a 10% sucrose solution provided ad libitum.

At 14 days postinfection (dpi), the female mosquitoes were immobilized using triethylamine (Sigma Aldrich, St. Louis, MO, USA). The mosquitoes were surface sterilized by dipping twice in 70% ethanol. To examine for ZIKV dissemination, the legs were removed from the mosquitoes and placed in individual tubes. Saliva was collected under sterile conditions by inserting the proboscis of the surface-sterilized female into a capillary tube containing ~ 20 µl filter sterilized fetal bovine serum plus 50% sucrose 1:1 for 30 min before subsequently ejecting the mixture into filter-sterilized 125 µl Mosquito Diluent [MD; 20% heat-inactivated fetal bovine serum in Dulbecco phosphate-buffered saline plus 50 µg/ml penicillin/streptomycin, 50 µg/ml gentamicin and 2 µg/ml Fungizone (Sigma Aldrich, St. Louis, MO, USA)]. As described in [39], briefly, to dissect the salivary glands, the tip of the abdomen was removed with a sharp cut using a dissecting probe. Placing a probe in the thorax, the head was removed gently by pulling away from the thorax. The salivary glands were then detached from the head using the probe, rinsed twice in sterilized phosphate-buffered Saline (PBS) (Sigma Aldrich, St. Louis, MO, USA), and transferred to a sterile microcentrifuge tube and stored at −80 °C. The midgut was pulled from the tip of the abdomen and rinsed twice in sterile PBS before being transferred to sterile microcentrifuge tubes and stored at −80 °C until tested.

The individual mosquito carcasses were then transferred into individual tubes sterile microcentrifuge tubes. All samples were held at −80 °C until assayed.

### Sequencing and analysis of microbiome of *Aedes* mosquito saliva and midguts

A ZIKV-specific quantitative PCR assay that targets the NS1 region [40] was utilized to obtain viral titer from the legs and saliva as described by [38]. The individuals whose midguts and saliva samples were infected with the virus were identified. ZIKV-infected saliva and midgut samples as well as those exposed to a naïve blood meal were used for downstream analysis.

We experienced challenges in obtaining enough genomic DNA material from singleton saliva samples and hence to increase the amount of genomic material for microbiome study, we adopted a whole transcriptome amplification approach. RNA was isolated from the saliva and midgut samples using Trizol (Thermo Fisher Scientific, Waltham, MA, USA). The saliva and midgut RNA samples were reverse transcribed, followed by whole transcriptome amplification according to the REPLI-g whole transcriptome amplification (WTA) single-cell kit protocol (Qiagen, Hilden, Germany). The resulting amplified complementary DNA (cDNA) was diluted 1:100. Thereafter, the 16S V3–V4 hypervariable region was amplified using an Illumina barcoded 16S primer set [16S_341F (TCG TCG GCA GCG TCA GAT GTG TAT AAG AGA CAG CCT ACG GGN GGC WGC AG) and 16S_805R(GTC TCG TGG GCT CGG AGA TCT GTA TAA GAG ACA GGA CTA CHV GGG TAT CTA ATC C)] [36]. PCR reactions were carried out in a total volume of 50 µl, 5 µM each of 16S ribosomal RNA (rRNA) primer pair, 2 µl WTA cDNA, 8 µl deionized filter-sterilized water, and 36 µl AccuStart II PCR supermix (Quantabio, Beverly, MA, USA). We included two negative controls at the PCR amplification step: water and nontemplate control. In addition, a positive spike at the sequencing step. A fragment size of  ~  460 bp of each sample was submitted to the Wadsworth Center Applied Genomics Core for sequencing together with the negative controls. Automated cluster generation and paired-end sequencing (250-bp reads) was performed on the Illumina MiSeq 500 cycle.

A total of 30 samples [five infected midguts (INF MG), six noninfected midguts (NINF MG), ten infected saliva (INF SAL), six noninfected saliva (NINF SAL), one negative control (NC), one nontemplate control (NTC), and one positive spike (PC)] were included in this study. Analysis of the data was carried out on QIITA (https://qiita.ucsd.edu/) and MicrobiomeAnalyst [41]. In summary, the command split libraries FASTQ was used to convert the de-multiplexed FASTQ files to the format used by QIITA. This was followed by clustering of the sequences into operational taxonomic units (OTUs), which was done by the utilization of a closed-reference OTU picking based on the GreenGenes 16S reference database. The choice of closed reference was due to its fully parallelizable process, which makes it a computationally efficient process. We neither elected to utilize open reference nor de novo OTU picking due to the computationally complex strategies that make them unsuitable for large data sets. Open-reference OTU picking reduces read loss by de novo clustering reads that do not match the reference sequences; some of the steps of the workflow run serially and hence slows down the process. De novo OTU picking, on the other hand, clusters reads against one another without an external reference collection, is computationally complex, results in large data set loss, and can reduce quantification accuracy while reducing the potential to detect rare taxa [42].

A 97% sequence identity threshold was utilized to assign taxonomy to the sequence. A BIOM-formatted OTU table was subsequently generated. To estimate alpha diversity metrics, we adjusted for differences in library sizes across samples using rarefaction and generated rarefaction curves to assess whether the sequencing depth was sufficient. Rarefaction involves picking a specified number of samples equal to or less than the number of samples in the smallest sample followed by a random removal of reads from larger samples until the number of remaining samples is equal to this threshold [43]. The reads have been deposited in NCBI GenBank Short Read Archives.

We anticipated the presence of rare taxa in our microbial community. To accommodate the rare taxa while estimating diversity, we utilized Chao1 diversity measure at the family taxonomic level to assess species richness of INF and NINF saliva and INF and NINF MG. Statistical significance was tested using a *t*-test assuming a normal distribution of values. Chao1 index was used to estimate alpha diversity because in estimating the diversity it makes inferences about rare or difficult-to-detect species [44]. We compared these results with other ecological indices used to test for alpha diversity such as the Shannon diversity and Simpson index. The Shannon diversity index considers both the richness and evenness of the microbial species present. It calculates this by utilizing the proportion of species *i* relative to the total number of species (*p*_*i*_) and multiplying by the natural logarithm of the proportion (ln*p*_*i*_) [45]. The Simpson Index tests for species dominance by considering the total number of species present as well as the relative abundance of each species. In this diversity index, species richness and evenness are directly proportional to diversity [46].

To compare the microbial community composition between the global INF and NINF saliva and INF and NINF MG, beta diversity was measured utilizing the Bray–Curtis dissimilarity statistic at the family taxonomic level [47]. The beta diversity measurement utilized the permutational analysis of variance (PERMANOVA) test to estimate the differences in microbial communities. PERMANOVA is a nonparametric multivariate statistical permutational test measured through a geometric partitioning of multivariate variation of a dissimilarity measure according to a given ANOVA design [48]. The distance matrix generated after comparing each sample to the other was visualized for the dissimilarity between samples using the principal coordinate analysis ordination method.

### Biomarker analysis

Random forest algorithm [49] within Microbiome Analyst [41] was utilized to identify taxa associated with the different infection statuses of saliva and midgut. Random forest algorithm [49] is a directed classification algorithm of trees generated by bootstrapping samples while training data and random feature selection in tree induction. It is an ensemble of unpruned classification or regression trees trained with the bagging method [50]. The default setting of the number of trees to grow and the number of predictors to try was applied (500 and 7, respectively) with the randomness setting left on. Further, we tried to adjust the parameters to different settings, but we did not observe any improvement in the model performance. We assessed the Out-of-Bag (OOB) error rate of the model.

## Results

### *Ae. albopictus* saliva contains a more diverse microbial profile than its midgut

Illumina MiSeq 16S rRNA sequencing effort resulted in 2,187,947 reads. A total of 936 OTUs were identified in this study (Supplementary File 1). The minimum number of reads observed was 31,362 and the maximum was 360,372 (Supplementary File 1). To adjust for differences in read depth, we rarefied our samples to a read depth of 31,632. The rarefaction curve analysis was performed, and the samples analyzed attained the saturation plateau, indicating that sequencing depth was sufficient (Supplementary Fig. 1).

**Fig. 1:**
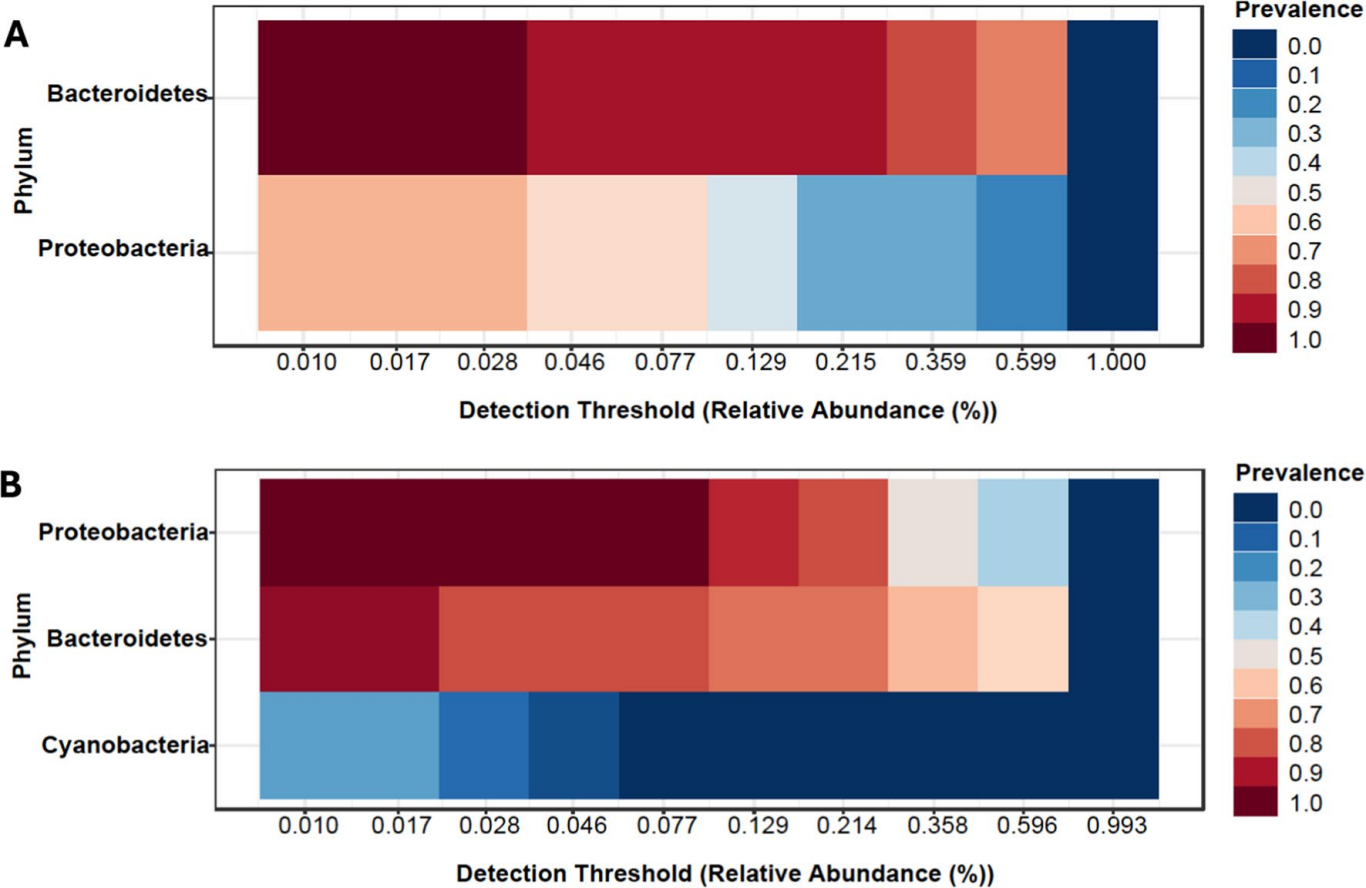
**A** The core phyla identified in the *Ae. albopictus* midgut were bacteria of the phyla *Bacteroidetes* and *proteobacteria*. **B** The *Ae. albopictus* saliva core phyla consisted of *proteobacteria*, *Bacteroidetes*, and *Cyanobacteria*

**Fig. 2:**
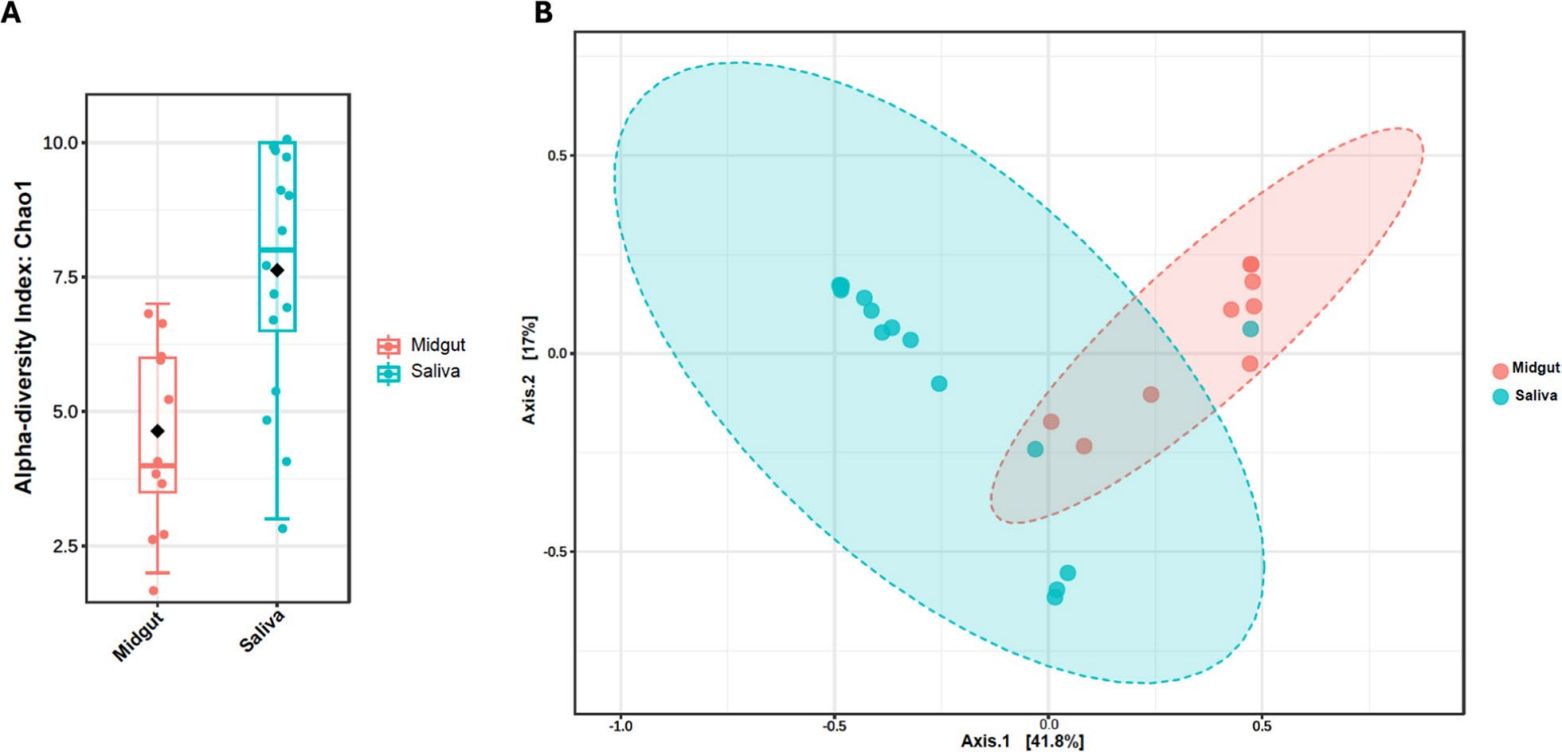
**A** Each box and whisker plot represents the variation in the microbial taxa in the midgut and saliva samples. The saliva samples have a richer microbial composition compared to the midgut (Chao 1; *P* = 0.0167; *t*-test statistic: −2.8268). **B** Principal component analyses were completed to assess the relationships among and between bacterial taxa identified in midgut and saliva. Despite several shared individual bacterial taxa, significant separation by tissue type was identified (PERMANOVA; *F*-value: 11.163; *R*^2^ = 0.3087; *P* < 0.001). The percentage of total variation explained by each axis is indicated

The core phyla of microbes identified in the midgut were bacteria of the phyla *Bacteroidetes* and *Proteobacteria* while that of saliva were Proteobacteria, Bacteroidetes, and Cyanobacteria (Fig. 1A, B). The *Ae. albopictus* saliva displayed a significant species richness compared with the midguts (Chao1; *P* = 0.0167; *t*-test statistic: −2.8268) (Fig. 2A). We extended our query of species richness to evenness, and this also demonstrated a diverse saliva microbial community (Shannon Index, *P* = 0.0013, *t*-test statistic: −3.7183; Simpson Index, *P* = 0.0012, *t*-test statistic: −3.7439) (Supplementary Figs. 2, 3). Both the saliva and midgut of the *Ae. albopictus* contained unique clusters of bacterial genera not shared between them, yet a few bacterial genera–*Elizabethkingia*, *Pseudomonas*, *Sphingomonas*, and *Wolbachia* were present in both the midgut and saliva samples (PERMANOVA; *F*-value: 11.163; *R*^2^ = 0.3087; *P* < 0.001; Fig. 2B; Supplementary File 2).

### ZIKV infection does not impact the midgut and saliva microbial diversity

A total of ten infected *Ae. albopictus* saliva and five infected midguts were analyzed in this study. An average viral RNA copies number range of log_10_ 1.05 to log_10_ 1.76 RNA copies/ml with a standard deviation of 0.22 were associated with the infected saliva samples, while an average viral RNA copies number range of log_10_ 1.47 to log_10_ 1.49 RNA copies/ml with a standard deviation of 0.008 were representative of the infected midgut samples.

**Fig. 3:**
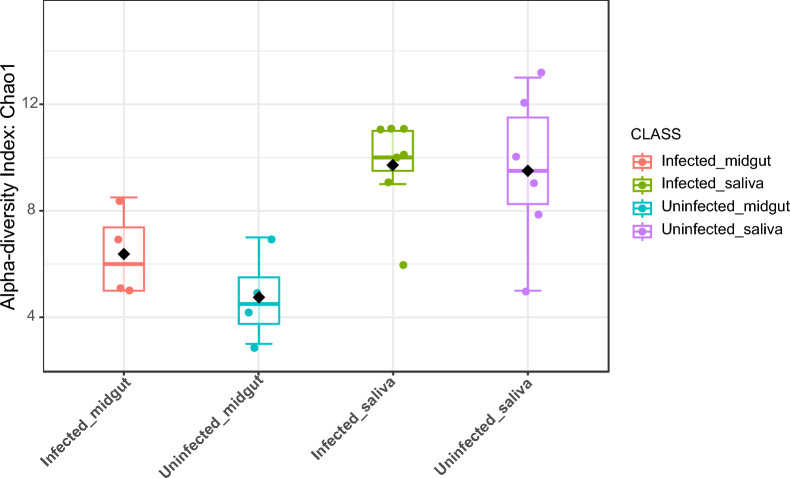
ZIKV infection resulted in a globally homogenous microbiome community among the INF and NINF saliva (Chao 1; *P* = 0.12749; ANOVA: *F* = 1.6755) and INF and NINF MG (Chao 1; *P* = 0.86761; ANOVA: *F* = −0.17159)

At a tissue-specific level, we observed that ZIKV infection neither altered the microbial diversity of the saliva (Chao1; *P* = 0.12749; [ANOVA]: F = 1.6755) nor the midgut (Chao1; *P* = 0.86761; [ANOVA]: F = −0.17159) (Fig. 3). Bacteria belonging to the *Elizabethkingia* spp. were significantly enriched in INF MG (average of 10 902 hits), NINF MG and NINF saliva had an average of 5696 and 1319 hits respectively while INF saliva had diminished levels (average of 22 hits) (ANOVA Test, *P* = 0.0003) (Supplementary File 2).

**Fig. 4:**
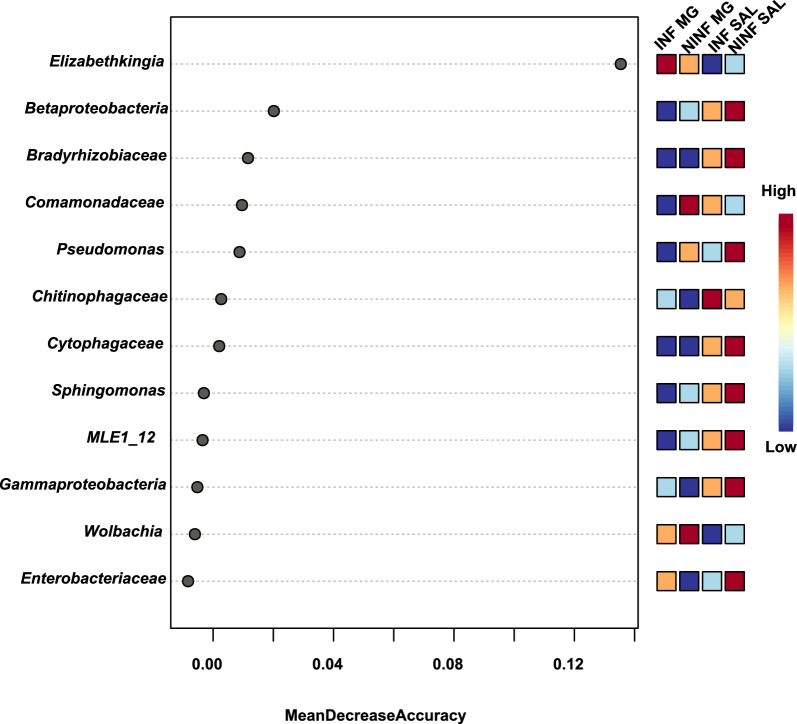
The Random Forest classifier was used to determine the association of bacterial taxa with the infection status of *Ae. albopictus* midgut and saliva. Mean decrease accuracy is reported for each of the taxa. This measure is obtained by removing the relationship of taxa and measuring the increase in error. The taxa with the highest mean decrease in accuracy is considered to have the highest association with the state. The highest mean decrease in accuracy was identified for *Elizabethkingia* associated with INF MG

The prediction error of the machine learning model of the random forest algorithm resulted in an Out-of-bag (OOB) error of 0.636. *Elizabethkingia* was most significantly associated with INF MG while *Wolbachia* was the most abundant taxa identified in the NINF MG. Bacteria belonging to the family Chitinophagaceae were found to be enriched in the INF SAL samples while the NINF SAL had several taxa associated with them (Fig. 4).

## Discussion

While microbes have been identified in mosquito salivary glands [51–53], there exist significant gaps in the study of mosquito saliva microbes. This is despite a few studies reporting the presence of microbes in the saliva of mosquitoes [20, 21] with the capacity to infect vertebrate host tissues and establish systemic infections. The current study aimed to compare the microbiome profile of *Ae. albopictus* saliva to its midgut. In addition, we studied the impact of ZIKV exposure on the saliva and midgut microbiome profile.

In this study, we observed that the core microbes in both saliva and midguts of *Ae. albopictus* consisted of *Bacteroidetes* and *Proteobacteria*. Our findings coincide with the findings of a study examining the microbial profile of saliva, salivary gland, and midgut tissues of *Anopheles gambiae* and *An*. *stephensi*. This study observed that similar taxa phyla were shared between the saliva, salivary glands, and midguts of *An. stephensi* with Proteobacteria as the main phylum. *An. gambiae* had two dominant populations in saliva and midguts, which were Proteobacteria and Bacteroidetes [20]. Our current study and the mentioned study utilized laboratory-reared mosquito strains, yet lab-reared mosquitoes are known to have an altered microbiome profile compared to wild populations [54]. Future studies utilizing freshly collected field samples will be important to compare with both findings [55].

Further, in this study, we observed a richer *Ae. albopictus* saliva microbiome compared with the midgut, yet several taxa were common to both the saliva and midguts. Previous study findings noted a more diverse bacterial taxon in the *An. gambiae* saliva compared with the midgut [20], as well as a richer and more complex microbial taxa in the salivary gland of naïve sugar-fed *An. culicifacies* compared with their gut [53]. We did not observe any alteration in richness in the microbiome community of the *Ae. albopictus* saliva exposed to ZIKV infectious blood meals. This corroborates the findings of studies of the impact of *P. berghei* infection on the *An. gambiae* and *An. stephensi* saliva microbial community [20]. In the future, it is important to study how the saliva microbiome interacts with arthropod-borne viruses present in the saliva. This is important as it will provide fresh insights into early activities at the skin bite site and may lead to novel approaches in designing transmission barrier tools [56].

Additionally, we did not measure a difference in richness in the midguts of individuals exposed to ZIKV infectious blood meal. These results differ from our previous findings which showed a significant reduction in *Ae. albopictus* midgut microbial diversity upon exposure to a ZIKV blood meal [34]. In addition, our findings are different from findings that demonstrate that *La Crosse orthobunyavirus* viral infection diminishes the midgut microbial diversity of *Ae. japonicus* and *Ae. triseriatus *[35]. The difference between our findings and the previous findings may be due to the differences in the experimental approach. Our current study included a genome amplification step in order to overcome limited starting genomic material of saliva, and this may have caused the differences observed. Future experimental approaches that overcome the barrier of limited genomic material will be important to ensure comparisons with past findings.

The Bray–Curtis dissimilarity statistic may have captured a portion of the variation in the microbial community; however, other factors such as differences in species richness, genetic diversity of the microbial taxa, and gene mutations may also explain the differences we observed. In the future, an all-encompassing study that included these other factors could provide a deeper understanding of microbial species diversity in mosquitoes.

We observed a significant increase in *Elizabethkingia* spp. levels in midguts associated with ZIKV-infected blood meal. In our previous findings, we have demonstrated that *E. anophelis* possesses an antiviral activity and accumulates in the midguts of *Ae. albopictus* associated with ZIKV infectious blood meals [34]. Further, our unpublished findings demonstrate that coinfection of ZIKV and *E. anophelis* in vitro results in a decrease in metabolites important for viral replication and progression of viral infection. Further, we observed the presence of *Elizabethkingia* spp. in the saliva of *Ae. albopictus* that were exposed to a naïve blood meal. The findings of this study corroborate a study that identified *Elizabethkingia* bacterial species in both the salivary glands and midguts of *An. Culicifacies *[53]. As well as the observation of *Elizabethkingia* spp. in the saliva of *An. gambiae* and *An. stephensi* after a naïve blood meal [20]. *E*. *anophelis* has been identified as a cause of sepsis as well as for neonatal meningitis [55]. By 2016, *E. anophelis* had caused outbreaks in the Midwestern USA that resulted in morbidity and mortality of  ~ 30% [55–57]. The Midwestern outbreak occurred primarily in community settings [56] and despite extensive investigations, no point source of infection has been identified [55, 57]. While the role of mosquitoes in the transmission of *E. anophelis* is unclear, our identification and previous study findings of *Elizabethkingia* spps. in mosquito saliva warrants the need for further studies to establish whether mosquitoes can transmit *E. anophelis* in the saliva. The findings of such a study will improve *E. anophelis* epidemiology and inform public health strategies.

One of the significant limitations of this study is the utilization of amplified RNA to overcome the significant challenge of minute amounts of starting genomic material. This approach may result in overamplification of the dominant bacterial species and underrepresentation of the rare bacterial taxa resulting in skewed results that may not be representative of the taxa diversity. Despite this possible limitation, we observed similar results as past findings of similar studies. In addition, by increasing the amount of starting material, we have been able to study microbial communities at an individual level. Future studies should consider pooling saliva samples to preclude any bias when studying saliva microbiome. Further, in this study, we worked with a small sample size, and our results may not provide a complete representation of the microbial composition of the midgut and saliva of *Ae. albopictus*. In the future, there is a need for a larger sample size and utility of wild *Aedes* spp. populations over lab-reared strains. Together, these findings demonstrate a need for further studies of aspects of mosquito saliva components that have not been studied in the past. This is important due to mosquito saliva’s primary role in blood acquisition and pathogen transmission.

## Conclusions

This study provides insights into the microbial community of the *Ae. albopictus* saliva and the impact of ZIKV infection on saliva and midgut microbial profiles. We observed a richer and more diverse saliva microbial community compared with the midgut. We identified *Elizabethkingia* spp., an emerging pathogen causing life-threatening infections in humans, in the saliva of *Ae. albopictus*. The results of this study lay a foundation for future exploratory studies on interactions of mosquito saliva microbiome and ZIKV to understand the role of mosquito saliva microbiome in ZIKV transmission. These findings can be utilized as an avenue for an innovative approach to develop a transmission-blocking tool. Lastly, the identification of *Elizabethkingia* spp., an emerging global threat causing infections with high mortality rates, is important and there is a need for a vector competence study to assess the capacity of *Ae. albopictus* to transmit the pathogen [58].

### Supplementary Information

Supplementary Material 1 File 1 Excel file showing the number of reads and OTU identified per sample.

Supplementary Material 2 File 2 Excel file showing the bacterial taxa associated with each saliva and midgut sample in this study.

Supplementary Material 3 Fig 1 Rarefaction curves of *Ae. albopictus* midgut and saliva showing sample sequence size on the *X* axis and species richness on the *Y* axis.

Supplementary Material 4 Fig 2 Box plot showing alpha diversity based on the Shannon Index.

Supplementary Material 5 Fig 3 Box plot showing alpha diversity based on Simpson Index.

## Data Availability

The dataset supporting the conclusions of this article is available in NCBI GenBank Short Read Archives under the accession numbers: PRJNA608493; PRJNA608495; PRJNA608496; PRJNA789914; PRJNA608499; PRJNA608500; PRJNA608501; PRJNA789908; PRJNA608416; PRJNA608418; PRJNA608503; PRJNA608489; PRJNA608483; PRJNA608482; PRJNA608427; PRJNA608428; PRJNA608472; PRJNA608479; SAMN11668157; SAMN11668158; SAMN11668159; PRJNA608480; PRJNA608481; PRJNA608505; SAMN11667562; SAMN11667607; SAMN1166775.

## Abbreviations

ZIKV: Zika virus
*rRNA*: Ribosomal RNA
DENV: Dengue virus
LTßR: Lymphotoxin β receptor
NYSDOH: New York State department of health
WCAGC: Wadsworth center applied genomics core
MD: Mosquito diluent
cDNA: Complementary DNA
INF MG: Infected midguts
NINF MG: Noninfected midguts
INF SAL: Infected saliva
NINF SAL: Noninfected saliva
NC: Negative control
NTC: Nontemplate control
PC: Positive spike
OTUs: Operational taxonomic units
