# Supplementary figures and images for: Aedes albopictus saliva contains a richer microbial community than the midgut
Source: Parasit Vectors. 2024 Jun 25;17:267. doi: 10.1186/s13071-024-06334-1 (PMC11197185; doi:10.1186/s13071-024-06334-1)

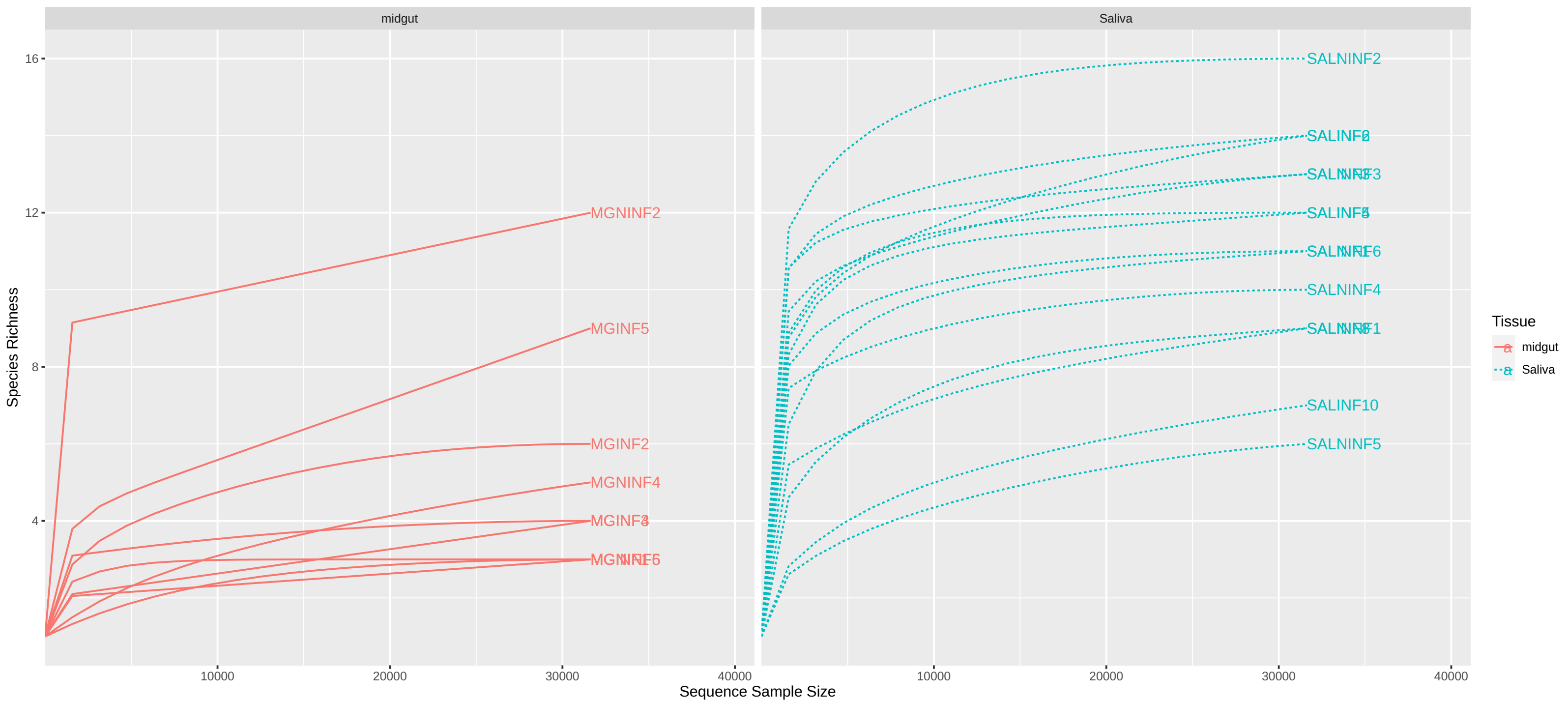

Supplement: Supplementary file 3 — Supplementary Material 3 Fig 1 Rarefaction curves of Ae. albopictus midgut and saliva showing sample sequence size on the X axis and species richness on the Y axis. [file 13071_2024_6334_MOESM3_ESM.pdf]

Alpha-diversity Index: Shannon

Midgut

Saliva

CLASS

Midgut

Saliva

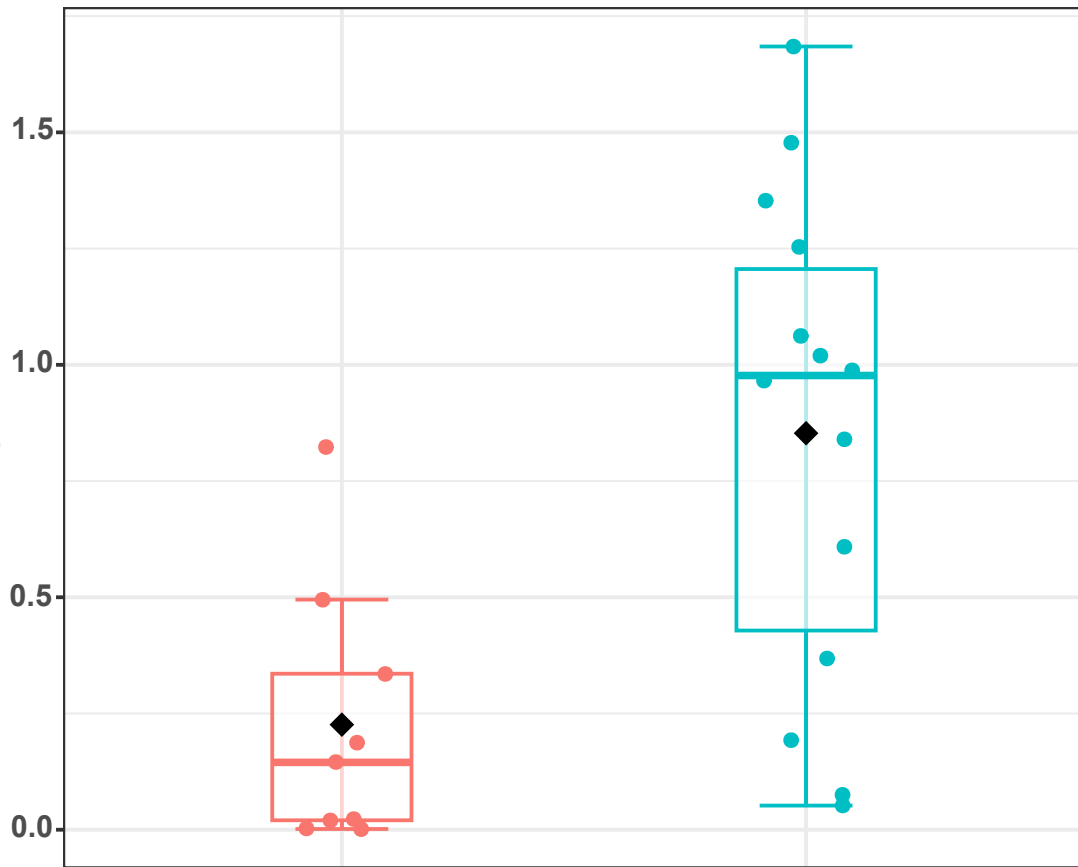

Supplement: Supplementary file 4 — Supplementary Material 4 Fig 2 Box plot showing alpha diversity based on the Shannon Index. [file 13071_2024_6334_MOESM4_ESM.pdf]

Alpha-diversity Index: Simpson

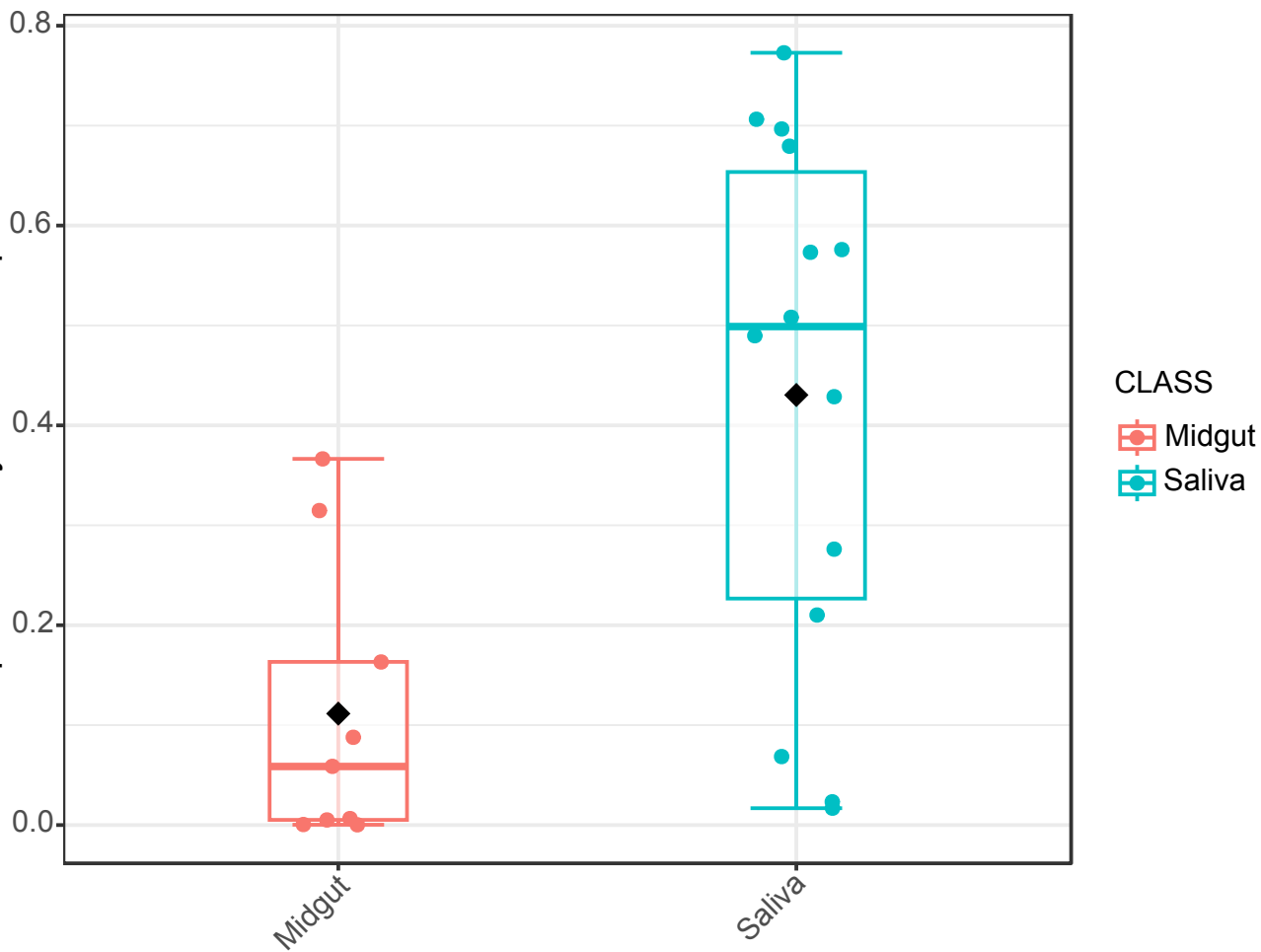

Supplement: Supplementary file 5 — Supplementary Material 5 Fig 3 Box plot showing alpha diversity based on Simpson Index. [file 13071_2024_6334_MOESM5_ESM.pdf]
